# Ionic liquid based surfactant-free microemulsion as a new protocol for preparation of visible light active poly(methyl methacrylate)/TiO_2_ nanocomposite

**DOI:** 10.1038/s41598-024-66872-7

**Published:** 2024-07-08

**Authors:** Alireza Salabat, Behnia Sadat Mirhoseini, Farid Mirhoseini

**Affiliations:** 1 Department of Chemistry, Faculty of Science, Arak University, Arak, 38156-8-8349 Iran; 2 Institue of Nanosciences and Nanotechnolgy, Arak University, Arak, 38156-8-8349 Iran

**Keywords:** Surfactant-free microemulsion, Polymer nanocomposite, Sensitized TiO_2_, Ionic liquid, Dye photodegradation, Nanoscience and technology, Pollution remediation

## Abstract

The practical application of sensitized TiO_2_ nanocomposites is very satisfying due to their high photon utilization in visible light, simple recovery without affecting the photocatalytic performance, high energy efficiency, low potential environmental risk, and low operational costs. The objective of this study is developing the ionic liquid (IL)-based surfactant-free microemulsion, as a soft template, for preparation of a novel type of sensitized poly(methyl methacrylate)/TiO_2_ nanocomposite (PMMA/TiO_2_/IL). For this purpose, a series of visible light-responsive PMMA/TiO_2_/IL transparent nanocomposites were prepared in microemulsion composed of methyl methacrylate monomer, 1-buthyl-3-methylimidazolium tetrafluoroborate ([bmim][BF_4_]), and 1-buthanol as amphi-solvent. Techniques such as diffuse reflectance spectroscopy (DRS))***,*** attenuated total reflectance-fourier transform infrared (ATR-FTIR) spectroscopy, atomic force microscopy (AFM), field emission scanning electron microscopy (FE-SEM), and energy dispersive X-ray analysis (EDX) were used to characterize prepared nanocomposites. Photocatalytic degradation of methyl orange dye under visible light illumination, as an application in wastewater treatment, with the investigation of the influence of TiO_2_ content in the nanocomposite, pH, and nanocomposite reusability on photodegradation efficiency was studied and maximum value of 93.9% obtained at optimum conditions. The FESEM analysis indicated that the utilization of a relatively low amount of ionic liquid and also in absence of the surfactant ensures the monodispersity of the visible light sensitized TiO_2_ nanoparticles in the polymer matrix.

## Introduction

Environmental pollution caused by the disposal of various chemicals in nature is not a new phenomenon and still carries the risk of illness and death for living organisms, including humans.

This phenomenon is increasing gradually and adversely affects the stability of the ecosystem and therefore has been the focus of attention for the last few decades^1,2^. Unfortunately, this phenomenon cannot be resolved through traditional strategies and conventional tools. To solve some part of this issue, developing and growing polymer photocatalytic technology as a green new approach is urgent and necessary^3–5^. At present, polymer nanocomposites based on visible light active photocatalysts for the removal of most environmental contaminations have attracted much attention because of environmentally friendly^6,7^.

For the past two decades, polymer-supported TiO_2_ nanocomposite or more precisely popular visible light sensitized photocatalyst nanocomposites have attracted much attention because this strategy avoids the need for costly post-treatment recovery, both time and money consuming^8,9^. Recently, Singh et al. prepared a series of nanocomposites based on the functionalized polymethylmethacrylate (PMMA) containing low dosage of TiO_2_ nanoparticles (1 wt% and 3 wt%) as antioxidant and antibacterial agents^10^. As a potential alternative to prevent filtration process, TiO_2_-based nanocomposites thin film have been prepared by Nicosia et al.^11^ They reported a recipe for preparation polymer nanocomposites having boosted activity for remediation xenobiotic water pollution.

One of the potent and elegant ways to furnish sensitized TiO_2_ nanoparticles in a polymer matrix with an engineered nano-shape is a microemulsion method^7^. An interesting microemulsion type can be formulated by ionic liquid (IL) to form IL-in-Oil (IL/O) or Oil-in-IL (O/IL) microemulsion as a soft template^12,13^. Microemulsions are, thermodynamically stable, isotropic and macroscopically homogeneous systems, including a polar phase (usually water) or hydrophilic ionic liquids (ILs), a non-polar phase (usually oil) or hydrophobic ionic liquids, and a surfactant.

Preparation of PMMA/IL visible light sensitized TiO_2_ nanocomposite with low dosage of TiO_2_ is a highlight topic in the polymer field researches^14–16^. In our first publication, the prepared PMMA/TiO_2_/IL nanocomposite by IL-based microemulsion method was used as a photocatalyst for methylene blue photodegradation and introduced for waste water treatment photocatalytic filter^13,17^. The role of the hydrophilic IL as polar phase in microemulsion system was investigated to produce visible light active polymer-photocatalyst nanocomposite. The other application of the prepared PMMA/TiO_2_/IL nanocomposite has also been investigated for degradation or photoreduction of 4-NP, trypan blue and MTBE^18,19^.

As mentioned in many papers, the use of several reactants is a main drawback for the fabrication of nanocomposites and other nanomaterials as the opposite of green chemistry principles. Recently surfactant free microemulsions (SFME), the opposite of the traditional surfactant-based microemulsions, formed in the absence of conventional ionic or nonionic surfactants have received considerable attention due to their some advantages such as low material cost, avoidance of ecotoxicity and simple purification^20,21^. SFME systems are usually composed of oil, water, and amphi-solvent. Principally the amphi-solvent in SFMEs formation should be completely or at least partially miscible in both oil and water phases. Up to now, the enzymatic reaction, chemical reactions, extraction, and nanomaterial synthesis have been explored and reported for potential applications of the SFMEs. A W/O SFME template-assisted hydrothermal method composed of hexane, propan-2-ol, and water for the preparation of hexagonal cylinder-like (twinning) ZnO microcrystals with excellent photocatalytic performance proposed by Zhang et al.^22^ As another application of SFMEs the preparation of hybrid adhesives based on poly(vinyl acetate) and commercial montmorillonite nanoclays were also investigated by Cazotti et al.^23^ They reported that the prepared PVAc/MMT hybrid latexes exhibited good colloidal stability and could be easily scaled-up to industrial conditions with improved properties.

In our previous research work, we prepared a new type of PMMA/Ag nanocomposite as an antibacterial agent by surfactant-free microemulsion consisting of methyl methacrylate (MMA) as the oil phase and 1-butanol as the amphi-solvent.^24^ The antibacterial activity of the prepared nanocomposites was also investigated and compared with nanocomposite prepared by traditional microemulsions.

In the current research work, the preliminary study about a new approach based on the ionic liquid in surfactant-free microemulsion to produce visible light active photocatalyst polymer nanocomposite with enhanced photocatalytic efficiency was investigated. The idea behind this work was a new combination of SFME microemulsion and a hydrophilic ionic liquid as polar domain in the SFME microemulsion (ionic liquid-in-oil) to produce the final nanocomposite product. Thus, the constituents of the SFMEs, especially applied ionic liquid for this study were chosen regarding the regulations in green chemistry principles because the ionic liquid can both disperse nanoparticles in microemulsion to form a stable TiO_2_ colloidal and also cause sensitization of them under visible light illumination.

As we know, the fabrication of PMMA/TiO_2_ film nanocomposite, as a novel photocatalyst system, by IL-in-oil surfactant-free microemulsion method has not been published to date. For this purpose, the IL-in-oil surfactant-free microemulsion route containing methyl methacrylate monomer as oil phase, hydrophilic IL of [bmim][BF_4_], as polar phase, and 1-butanol as amphi-solvent was used to fabricate PMMA/TiO_2_/IL nanocomposite. Before preparing the polymer nanocomposites, the phase diagram of the ternary system was studied and the microemulsion region in the phase diagram has been determined. Then a stable SFME formulation in a single-phase microemulsion region was chosen to fabricate nanocomposites. As mentioned above, the [bmim][BF_4_], was employed to stabilize TiO_2_ nanoparticles in the suggested formulation, and a series of SFME systems with different TiO_2_ dosages were prepared. After that, benzoyl peroxide (BPO) as initiator was added to start the polymerization and produce the visible light active PMMA/TiO_2_/IL nanocomposites. The synthesized nanocomposites were characterized using diffuse reflectance spectroscopy (DRS) ), attenuated total reflectance-fourier transform infrared (**ATR**-**FTIR**) spectroscopy, atomic force microscopy (AFM), field emission scanning electron microscopy (FE-SEM), and energy dispersive X-ray analysis (EDX) along with elemental mappings. The application of the novel modified PMMA/TiO_2_/IL nanocomposites in photodegradation of MO dye under visible light irradiation was also investigated. The effects of some factors on the photodegradation of MO dye such as TiO_2_ loading, pH, and recycling of the nanocomposite with the kinetics of the photodegradation reaction were studied.

## Experimental procedures

### Materials

1-butyl-3-methylimidazolium tetrafluoroborate ionic liquid ([bmim][BF_4_]) (≥ 98.0%) as polar phase was obtained from Sigma-Aldrich. Titanium dioxide (TiO_2_) nanoparticles were Degussa P25 (ca. 80% anatase and 20% rutile) with a BET surface area of 50 m^2^/g and particle size of less than 15 nm. Monomer of methyl methacrylate (AR grade) as oil phase without purification (99%), hydrophobic initiator of benzoyl peroxide (BPO), methyl orange (MO) dye and 1-butanol (99.5%), were received from Merck. NaOH and HCl for adjusting pH value were analytical grade reagents. Triple-distilled water with a specific conductivity of about 0.05 µS.cm^−1^, was used to prepare all solutions.

### Phase diagram construction

According to our previous experience in the investigation of SFME system containing MMA as oil phase, 1-butanol as amphi-solvent, and water, the direct observation method was also used for determining phase diagram of the microemulsion system containing [bmim][BF_4_] instead of water^24^. In this method 1 mL of the binary mixtures of MMA and [bmim][BF_4_] with specified mass ratio of MMA to IL (R_O/IL_) from 1:94 to 94:1 was prepared in a series of test tubes, and put into the water bath at 298.15 K with constant stirring. These solutions were titrated separately with 1-butanol as the third component, as long as the mixtures become clear by visual observation (transition from turbid to clear). The clear and transparent systems can be considered microemulsion and turbid systems were considered as multiphase. The entire procedure was repeated three times for all samples and the average volume of 1-butanol was noted. Subsequently, the percentage of each components of 1-butanol, IL and MMA was calculated, the boundary of ME-forming determined and pseudo-ternary phase diagrams were generated using Tri-plot software version 4.1.2.

### *Preparation of IL-in-oil microemulsions containing TiO*_*2*_

Different loading of TiO_2_ nanoparticles in IL/MMA (IL/O) SFME microemulsion systems were formed as follows. In the first step a certain amount of TiO_2_ nanoparticles added to IL and ultrasonically dispersed until TiO_2_/IL homogeneous mixture was obtained. In the second step the MMA was added to the TiO_2_/IL homogeneous mixture and mixed for 20 min by stirring. The obtained mixture at this step was cloudy. The obtained mixture was titrated by 1-buthanol until formation of a clear SFME microemulsion at room temperature. A transparent and stable over months of IL/MMA SFME microemulsion system consisting of TiO_2_ nanoparticles was obtained. As the polymerization temperature of MMA was 60°C, the stability of the microemulsion at this temperature system also confirmed.

### Preparation of the nanocomposites

In this step, to start the polymerization procedure, the BPO initiator (0.2 wt.% based on the weight of MMA) was added to the obtained SFME microemulsion systems in the previous section. All of the SFME microemulsion systems were kept at 60 °C under static condition for 8 h. Five nanocomposite samples were prepared at different TiO_2_ dosages, ranging from 0.0 wt.% to 0.012 wt.%. The samples with TiO_2_ weight percent of 0.0, 0.006, 0.008, 0.010, and 0.012 were marked as S0 (for pure PMMA), S1, S2, S3, and S4, respectively.

### *Characterization of the prepared PMMA/TiO*_*2*_*/IL nanocomposites*

The band gap energies of the nanocomposite samples were determined to confirm their visible light photocatalytic activity. For this purpose the cut-off wavelengths were obtained, by linear extrapolation from the inflection point of the curve to baseline with UV–visible diffuse reflectance spectra (UV–vis/DRS) in the wavelength range of 200–600 nm by using a spectrophotometer of JASCO, V-670.

The field emission scanning electron microscopy (FE-SEM) was used to investigate the nanocomposites morphology. The energy dispersive X-ray analysis (EDX) was also performed to indicate elemental analysis of the prepared nanocomposites. The FE-SEM instrument was equipped with an energy dispersive X-ray spectrometer (TESCAN MIRA3).

Attenuated total reflectance-Fourier transform infrared (ATR-FTIR) spectroscopy was conducted on a Bruker ALPHA FTIR Spectrometer.

To investigate the surface morphology of the nanocomposite sample the atomic force microscopy (AFM) with AFM (BRISK) was used.

### *Photocatalytic activity of the prepared PMMA/TiO*_*2*_*/IL nanocomposites*

**Figure 1:**
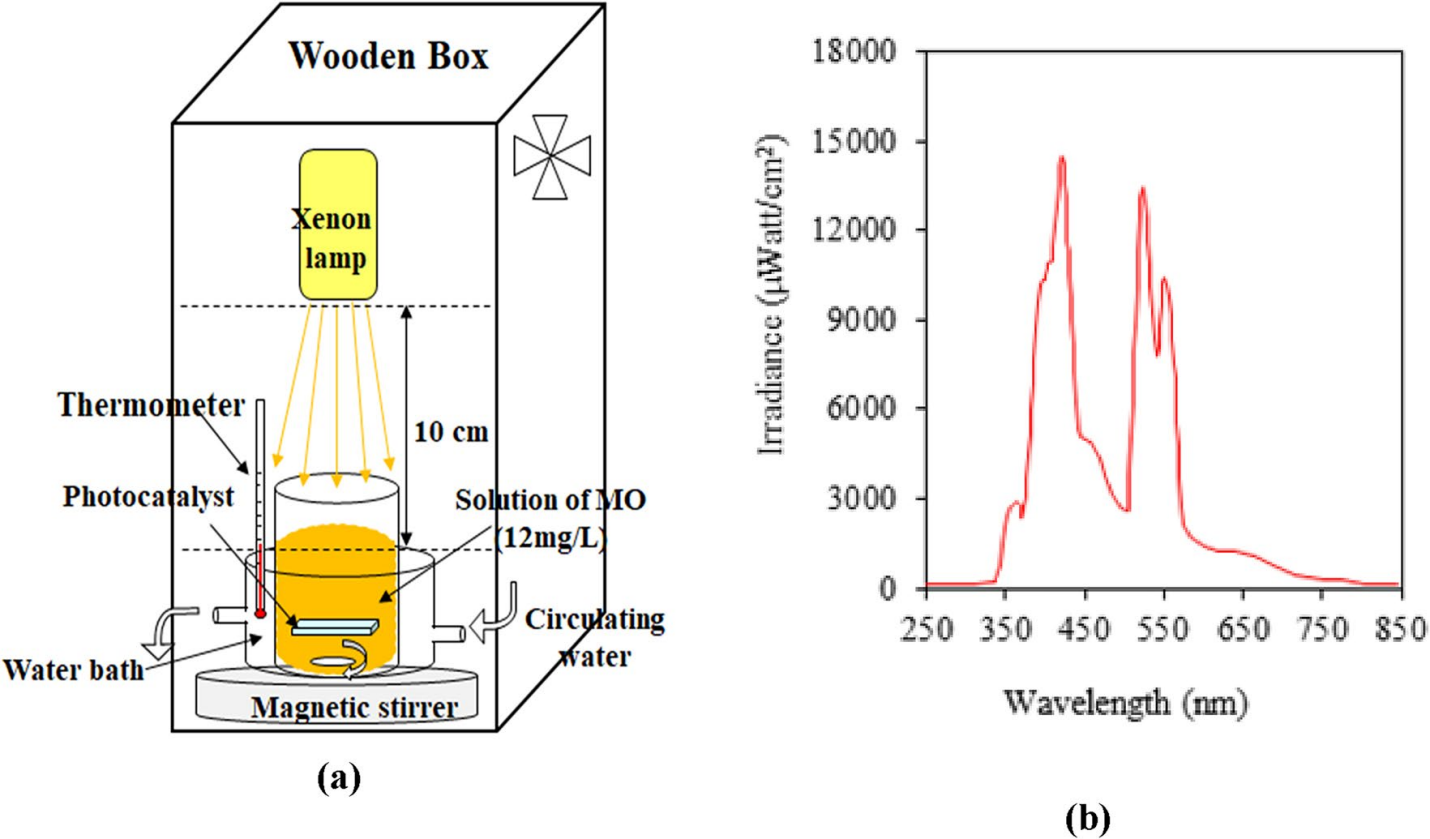
(**a**) The photocatalytic setup, and (**b**) the emission spectrum of the utilized xenon lamp as a visible light source in experimental setup in MO photodegradation.

As per our previous experience in water pollutants photodegradation assessment, a photoreactor setup, as shown in Fig. 1a was used for photodegradation of MO, dye as an azo dye^17–19^. In this study, a glass beaker as photoreactor that equipped with a magnetic stirrer and thermostatic bath was used with the aid of a xenon lamp (55 W) as the visible light source, positioned at the top of the photoreactor. The distance between the light source and the beaker reactor was fixed at 10 cm. The emission spectrum of the xenon lamp, taken using the Avaspec 2048 TEC instrument is shown in Fig. 1b. The reaction solution was prepared by addition of each PMMA/TiO_2_/IL nanocomposite samples to 15 ml MO aqueous solution with a concentration of 4 × 10^–5^ M or (12 ppm). Firstly, the solutions were maintained in dark condition for 10 min prior to exposure to the light and commencing illumination, to establish an adsorption–desorption equilibrium at 25 °C. After that, the MO solutions irradiation started and 5 ml of the reaction solution was sampled at given time intervals (every 30 min) to quantify the concentration of residual dye in the supernatant solutions, by UV–Vis spectrophotometric method, for a total photodegradation time of 6 h. The photodegradation percentage was calculated using the following equation:

1 $$\text{Photodegradation }(\text{\%}) =\frac{{A}_{0}-{A}_{t}}{{A}_{0}}\times 100$$

where *A*_*o*_ is the initial concentration of MO dye in the solution at zero time and *A*_*t*_ is the concentration of MO dye in the solution at time *t*. The photodegradation rate and kinetics were calculated by plotting *ln A*_*0*_*/A* versus times based on the first-order reaction according to Eq. (2). *k* represents the apparent rate constant (min^−1^).

2 $$ln\frac{{A}_{0}}{A}=kt$$

Additionally, the visible light photocatalytic degradation of MO dye was also performed against all three blank reactions with MO self-photolysis, bare TiO_2_ powder, and pure PMMA under the same process.

The stability and reusability of the heterogeneous nanocomposites are their main characteristics of them. To examine the stability and reusability, a nanocomposite was chosen that showed the best MO photocatalytic degradation. The recycling experiments were performed for six consecutive cycles. After each run, the polymer nanocomposite was removed from the colorless solution, and without any treatment (such as washing and drying) was utilized for the next test.

## Results and discussion

### Phase diagram of the ionic liquid based SFME system

**Figure 2:**
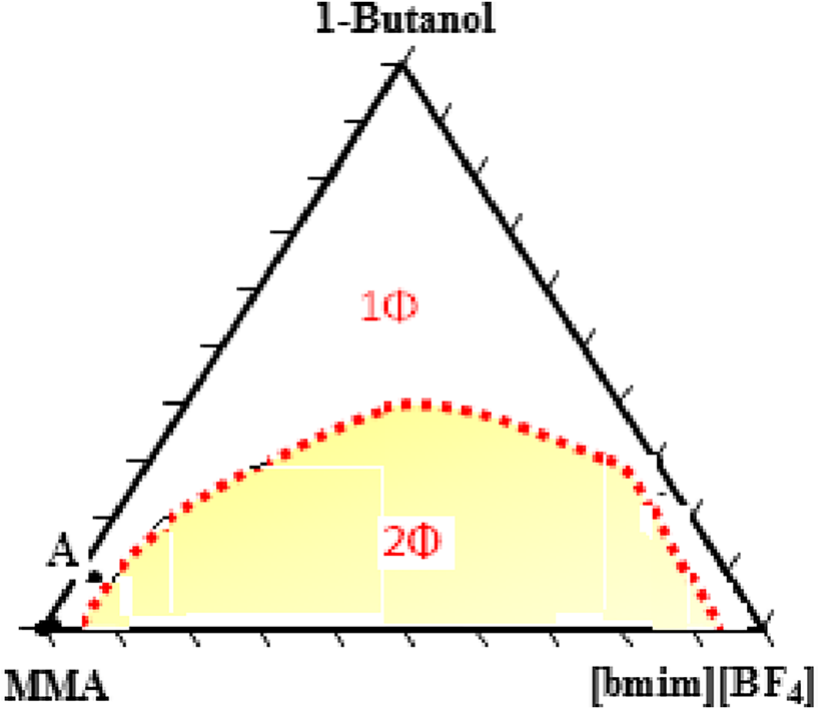
Phase diagram of the MMA/[bmim][BF_4_]/1- butanol ternary system at 298.15 K. Point (A) in the IL-in-oil microemulsion region was chosen for the preparation of nanocomposite**.**

The prepared triangular phase diagram of the ionic liquid based SFME system containing MMA, [bmim][BF_4_] and 1- butanol at 298.15 K is shown in Fig. 2. As can be seen, the single-phase of the surfactant free microemulsion domain (1Φ) extended from the [bmim][BF_4_] corner to near the MMA oil-rich corner and was verified visually on the basis of transparency and the absence of phase separation. It is clear that the area of the single and also two-phase region is greatly different for microemulsion systems due to the difference in composition and occupies a different area in the total phase. Based on the diagram phase the composition of point (A), as a stable formulation in the IL-in-oil region, composed of 88 wt.% of MMA, 10 wt.% of 1-butanol and 2.264 wt.% of IL was formed for nanocomposite preparation.

### Characterization results of the nanocomposites

The prepared nanocomposites were characterized using DRS, FE-SEM, and EDX methods and results discussed in the following.

### DRS results

**Figure 3:**
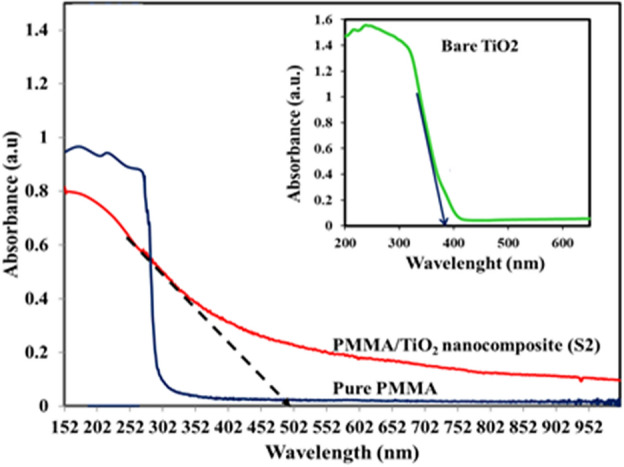
UV–vis diffuses reflectance spectra of the TiO_2_ nanoparticles, PMMA and PMMA/TiO_2_ nanocomposite.

DRS results of pure PMMA, and PMMA/TiO_2_ nanocomposite sample (S2), as a typical sample, which was prepared in the IL/O surfactant-free microemulsion system, are presented in Fig. 3. In this figure, DRS of the bare TiO_2_ is also shown as inset. From the optical absorbance data for all three samples the band gap energies were calculated by using the following equation.

3 $${E}_{g}=\frac{1239.8}{\lambda }$$

In this equation *E*_*g*_ is the band gap energy (*eV*) and* λ* is the wavelength of the absorption edges or cut-off wavelength (nm). Practically, the cut-off wavelength was determined by linear extrapolation from the inflection point of the curve to baseline as shown in Fig. 3. As can be seen the absorbance of the bare TiO_2_ sample is about 400 nm and band gap was obtained 3.20 eV. For PMMA/TiO_2_ sample fabricated in the IL/O surfactant-free microemulsion the absorbance wavelength was obtained more than 400 nm (about 502 nm) and displays an obvious red-shift into the visible-light region, and then its band gap calculated as 2.46 eV. It is evident that the absorption edge shifting related to the presence of the IL molecules, which are used as a substitute for the polar phase to form a novel ionic liquid-based surfactant-free microemulsion system and consequently the interaction of IL molecules and TiO_2_ nanoparticles. In comparison with PMMA/TiO_2_ sample fabricated in the IL/O surfactant-based microemulsion (Eg = 2.55 eV), the sample prepared in surfactant-free microemulsion exhibited a higher absorption in the visible region and a little more shifted toward the red light^13^. This small difference between the bandgap of PMMA/TiO_2_ nanocomposites prepared by two methods including surfactant-free microemulsion and surfactant-based microemulsion may be is induced by the presence of the TX-100 surfactant. It can be concluded that in the presence of the TX-100 surfactant, weak interaction of the TiO_2_ surface and imidazolium cation of IL is carried out.

### FE-SEM micrographs and EDX analysis

**Figure 4:**
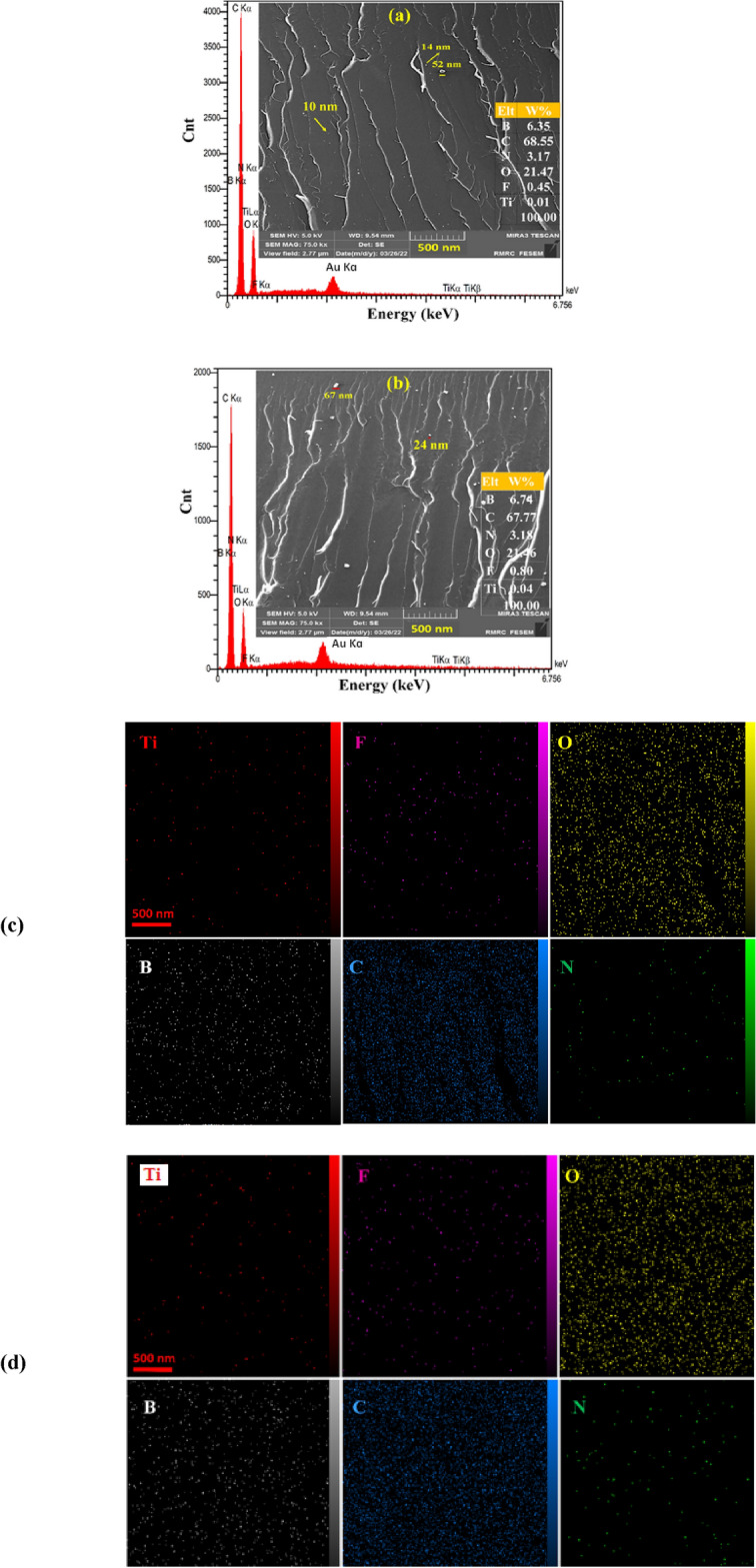
The FE-SEM images, EDX analysis ((**a**) S2 and, (**b**) S3 samples) with elemental mappings of PMMA/TiO_2_/IL nanocomposites ((**c**) S2 and, (**d**) S3 samples).

FE-SEM can provide a direct image of the activated TiO_2_ nanoparticles, the aggregates, their morphology, and also distribution in the polymer matrix of the fractured surface of nanocomposites. Figure 4 displays the FE-SEM micrographs and their EDX analysis along with elemental mappings for two samples S2 and S3 of PMMA/TiO2/IL nanocomposites. The elemental analyses of the nanocomposites by EDX shows typical peaks namely C, O, N, B, Ti, and Au. The peak of F signature from ionic liquid located at 0.64 eV and also peaks of B and N from imidazolium cation can be observed at around 0.20 eV and 0.43 eV, respectively. As can be seen in the EDX results, the intensity of the Ti peaks increased with increasing TiO_2_ content in the nanocomposite. The elemental mapping for two samples of S2 and S3 has been shown in Fig. 4c and d, respectively.

This result indicates that TiO_2_ particles don’t get aggregated and show good monodispersity and a fine morphology with homogenous and uniform particle size in PMMA. As can be seen, Fig. 4a shows a smooth surface for S2 sample with an average particle size of 10–14 nm compared to S3 sample with an average particle size of 14–26 nm with the most popular particles of 50–70 nm (Fig. 4b). The obtained results show that the IL, as a good dispersant and sensitizing agent due to the existence of huge organic imidazolium cations, has the main role to disperse sensitized TiO_2_ nanoparticles and there is a fine interaction with adsorbed IL molecules and the surface of TiO_2_ nanoparticles to have good compatibility with PMMA.

### (ATR)-FTIR study

**Figure 5:**
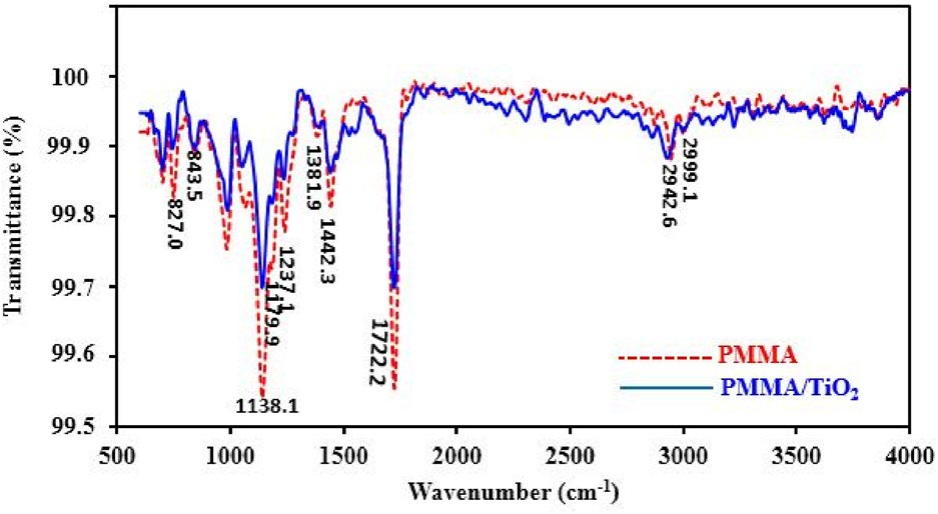
ATR-FTIR of pure PMMA and S2 nanocomposite sample.

As shown in Fig. 5, the IR band appeared at 500–850 cm^−1^ is related to the Ti–O–Ti stretching vibration mode in the TiO_2_ crystal. The major features of the pure PMMA spectra such as the C=O ester carbonyl stretching vibration mode (1722 cm^−1^), methyl, ester-methyl, and methylene C–H stretching in C–CH_3_ (2800–3100 cm^−1^) and bending (1350–1500 cm^−1^) mode were detected. Three bands in the 1100–1350 cm^−1^ region of the spectrum, which have been assigned to ester group stretching vibrations or the coupled C–O and antisymmetric C–C–O stretch (1237 cm^−1^) and skeletal vibrations coupled to C–H deformations (1179 and 1138 cm^−1^) were also detected. The band at 843 cm^−1^ is assigned to the methylene rocking mode, whereas the latter two bands at 827 and 809 cm^−1^ are associated with vibrations of the ester group, possibly the C–O–C symmetric stretching mode.

As can be seen from the spectrum for S2 nanocomposite sample, all characteristic PMMA backbone peaks are also observed in the PMMA/TiO_2_ nanocomposite. But by the presence of TiO_2_ nanoparticles in the PMMA polymer matrix, these bands were all observed to be altered and a slight shift with strong intensity reduction in main peaks were founded. For example, a little shift on 1722 cm^−1^ to 1721 cm^−1^, which may be attributed to the interaction between the carbonyl group and surface of TiO_2_ for carbonyl stretching vibration mode was happened, when TiO_2_ nanoparticles were immobilized in PMMA and pure PMMA changed to a PMMA/TiO_2_ nanocomposite. Thus, it is evident that there is chemical interaction during the in-situ polymerization of MMA monomer in presence of ionic liquid sensitized TiO_2_ nanoparticles.

### AFM result

**Figure 6:**
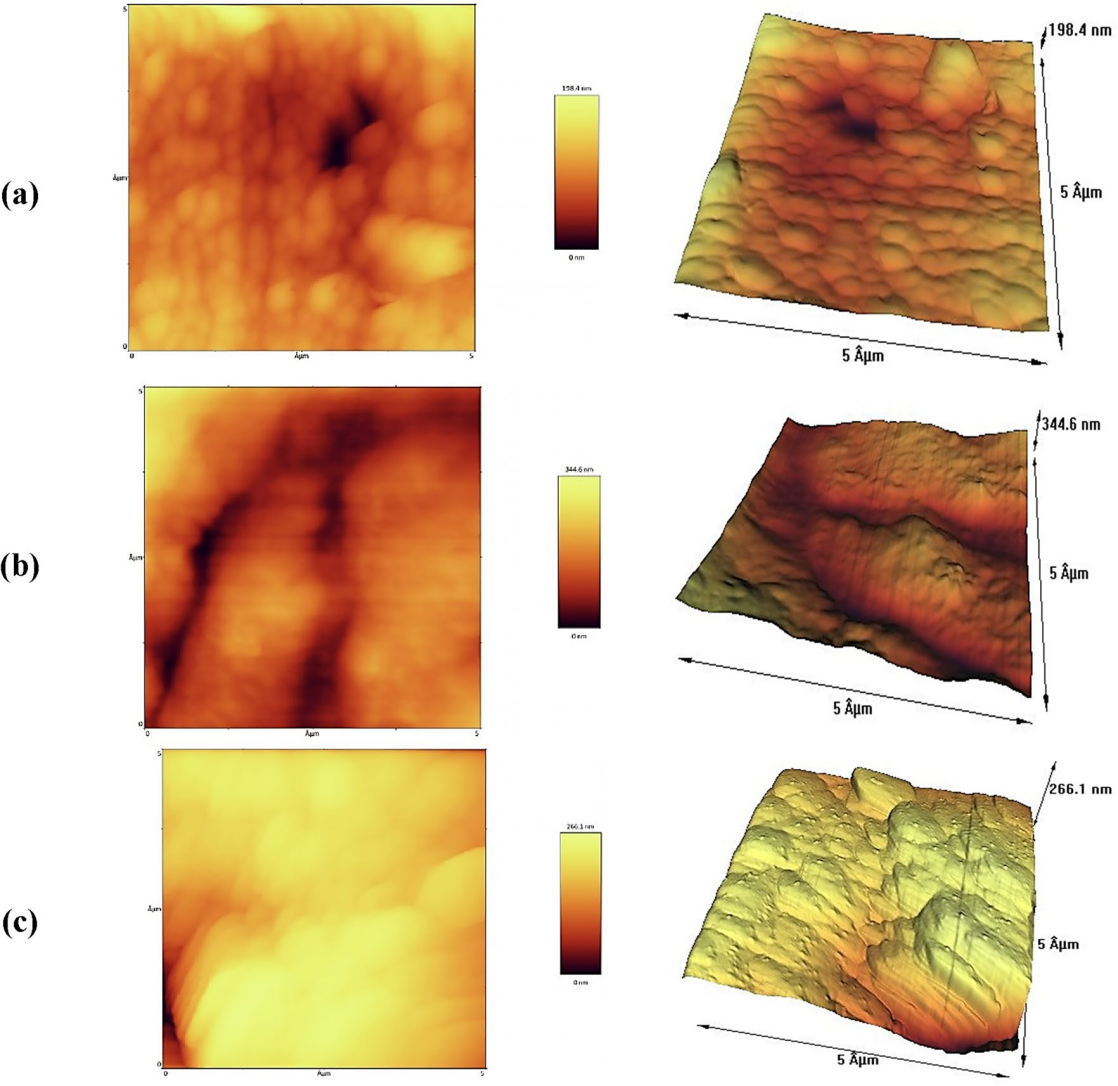
AFM images of the surface morphology of (**a**) pure PMMA, (**b**) S2 nanocomposite sample and (c) cross-section cut of the S2 nanocomposite sample.

The AFM photographs of the pure PMMA and the PMMA/TiO_2_ nanocomposite (S2 sample) are presented in Fig. 6a and b, respectively. A cross-section cut of the S2 nanocomposite sample was also carried out and presented in Fig. 6c.

The PMMA is exhibited the uniform and regular aspect on the amplitude and height. The slight roughness of the surface appearance may be due to the formation of small bubbles during polymerization. In comparison with pure PMMA, regarding the S2 sample containing a low loading of TiO_2_ nanofillers, it was difficult to catch the “face” of TiO_2_ nanoparticles on the surface image of polymer nanocomposite, which may be attributed to the excellent dispersion and existence of nanoparticles inside the polymer nanocomposites under this concentration. However, upon examining the cross-section surface of the S2 nanocomposite, the TiO_2_ nanoparticles in more regions was observed. This was evident from the height image, which showed that the nanoparticles were uniformly distributed into the polymer substrate with high monodispersity (Fig. 6c).

### Photocatalytic results

In this section, to evaluate the photocatalytic behavior of all prepared visible light active nanocomposites, the photodecolorization of the MO aqueous solution under visible irradiation at natural pH of aqueous MO solution (pH = 6.5) was investigated.

### *Effect of TiO*_*2*_* dosage on the photodegradation of MO*

In order to investigate the effect of TiO_2_ content of nanocomposites on the photodecolorization of MO aqueous solution (12 ppm) under visible irradiation, the experiment has been done for all nanocomposite samples (S1 to S4), bare TiO_2_ and pure PMMA. The photocatalytic activity of all experiment samples has been identified by determining the change in the typical absorption peak of MO at 464 nm in the absorption spectra of the samples at various time intervals.

**Figure 7:**
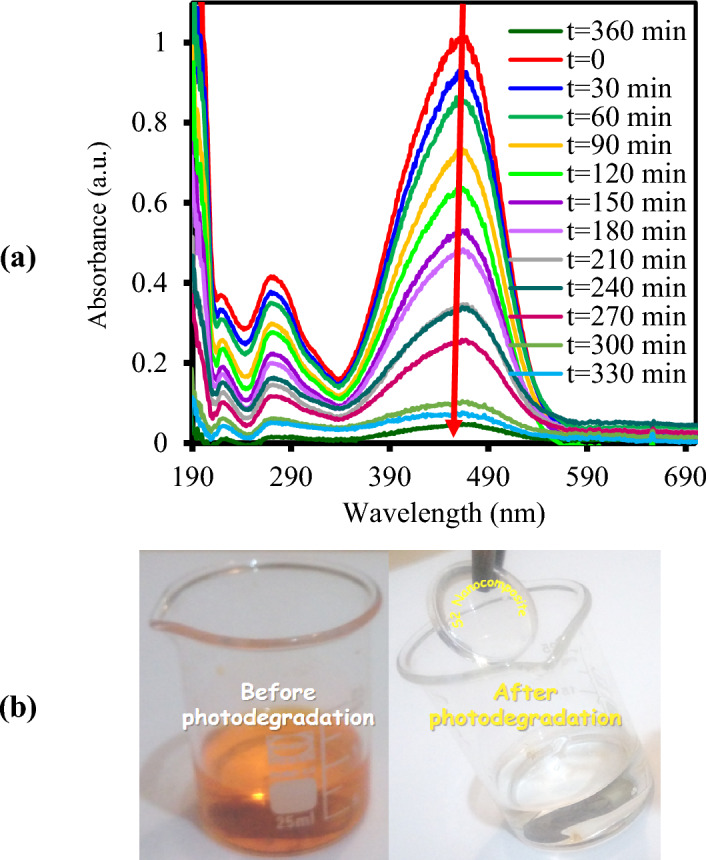
**(a**) Spectral patterns of MO dye solution and (**b**) MO degradation during the photocatalytic reaction in the presence of PMMA/TiO_2_/IL (S2) nanocomposite under visible-light irradiation for 6 h.

Figure 7a gives the MO solution absorption spectra changes exposed to visible light irradiation at regular time periods for a total time of 6 h with the nanocomposite sample of S2. The solution color diminished gradually from orange into very light orange with the irradiation time increasing during the photocatalytic experiment over the nanocomposite, and finally changed to colorless solution (Fig. 7b). It is verified that both catalyst and light irradiation are essential to photocatalytic activity and no photocatalytic performance was observed for MO self-photolysis, and pure PMMA. The photodecolorization of MO approaches about 22% using pristine TiO_2_ after 360 min and it should be related to the UV part of the employed Xenon lamp (Fig. 1b). The photodegradation percentage (Eq. 1) and first order rate constants photodegradation reaction (Eq. 2) over PMMA/TiO_2_/IL with different TiO_2_ dosage were calculated and reported in Table 1.

**Table 1:** The photodegradation percentage and first order rate constants of MO photodegradation reaction over PMMA/TiO_2_/IL with different TiO_2_ dosage.

Sample	*k* × 10^–3^ (min^−1^)	R^2^	Degradation of MO after 360 min (%)
MO self-photolysis	0.4	0.9986	3.1
PMMA	0.6	0.9943	4.2
Bare TiO_2_	1.6	0.9924	22.4
S1	1.7	0.9913	48.6
S2	4.7	0.9746	83.9
S3	4.4	0.9838	80.0
S4	4.2	0.9926	78.7

**Figure 8:**
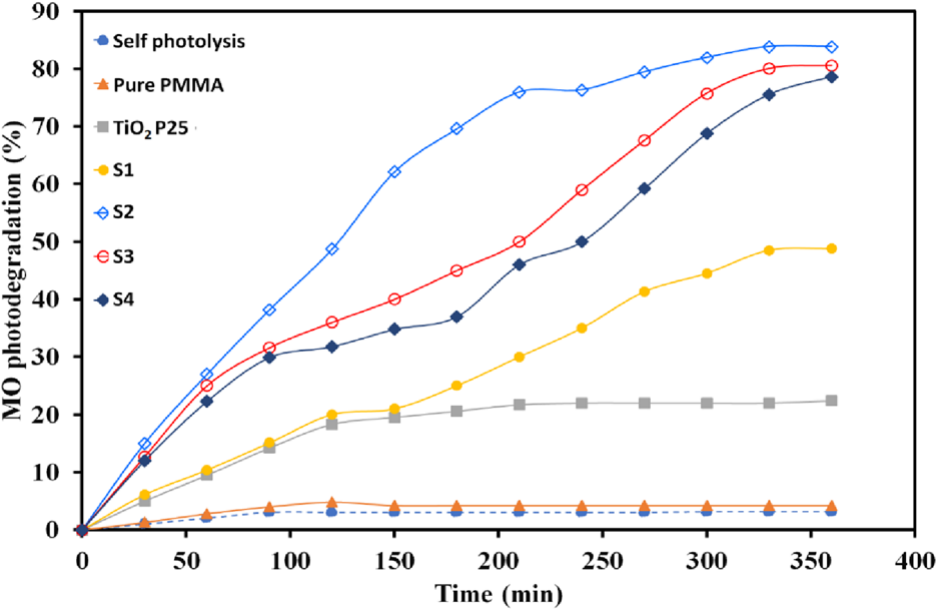
The photodegradation percentage of MO without nanocomposite (self- photolysis) and over pure PMMA, TiO_2_, PMMA/TiO_2_/IL with different TiO_2_ dosage.

As seen from Fig. 8 and Table 1, with increasing TiO_2_ loading in the polymer matrix, the MO photodegradation first increases from S1 to S2 nanocomposite, and with further increasing of TiO_2_ dosage, the photocatalytic performance decreases a little for S3 and S4. The photocatalytic activity for all samples at the same condition was determined in the following order: self-photolysis ≈PMMA < bare TiO_2_ < S1 < S2 > S3 ≈ S4. The maximum MO photodegradation efficiency and rate constant, *k* was about 83% and 0.0047 min^−1^, respectively, belonging to sample S2 of PMMA/TiO_2_/IL nanocomposite at pH = . This trend may be related to the agglomeration of the TiO_2_ nanoparticles with increasing concentration and reducing effective surface of nanoparticles and consequently low visible absorption can act as recombination centers of electrons and holes^25,26^.

In our previous reports for the PMMA/TiO_2_/IL nanocomposite that was prepared by surfactant based microemulsion, sample containing 0.01 wt.% of TiO_2_ exhibited the best performance^13^. In the current study, PMMA/TiO_2_/IL nanocomposite including 0.008 wt.% of TiO_2_ showed the more efficient photocatalytic activity for MO degradation. This phenomenon may be related to the role of surfactant to block the effective sites of the photocatalyst. Chai et al.^27^ prepared the TiO_2_ nanoparticles by the surfactant-free microemulsion–hydrothermal method at lower temperatures using oil-in-water SFME systems as templates. They reported that the TiO_2_ nanoparticles, which avoid the blocking effects of surfactant molecules on the active sites of the nanoparticles, exhibited the highest photocatalytic activity.

**Figure 9:**
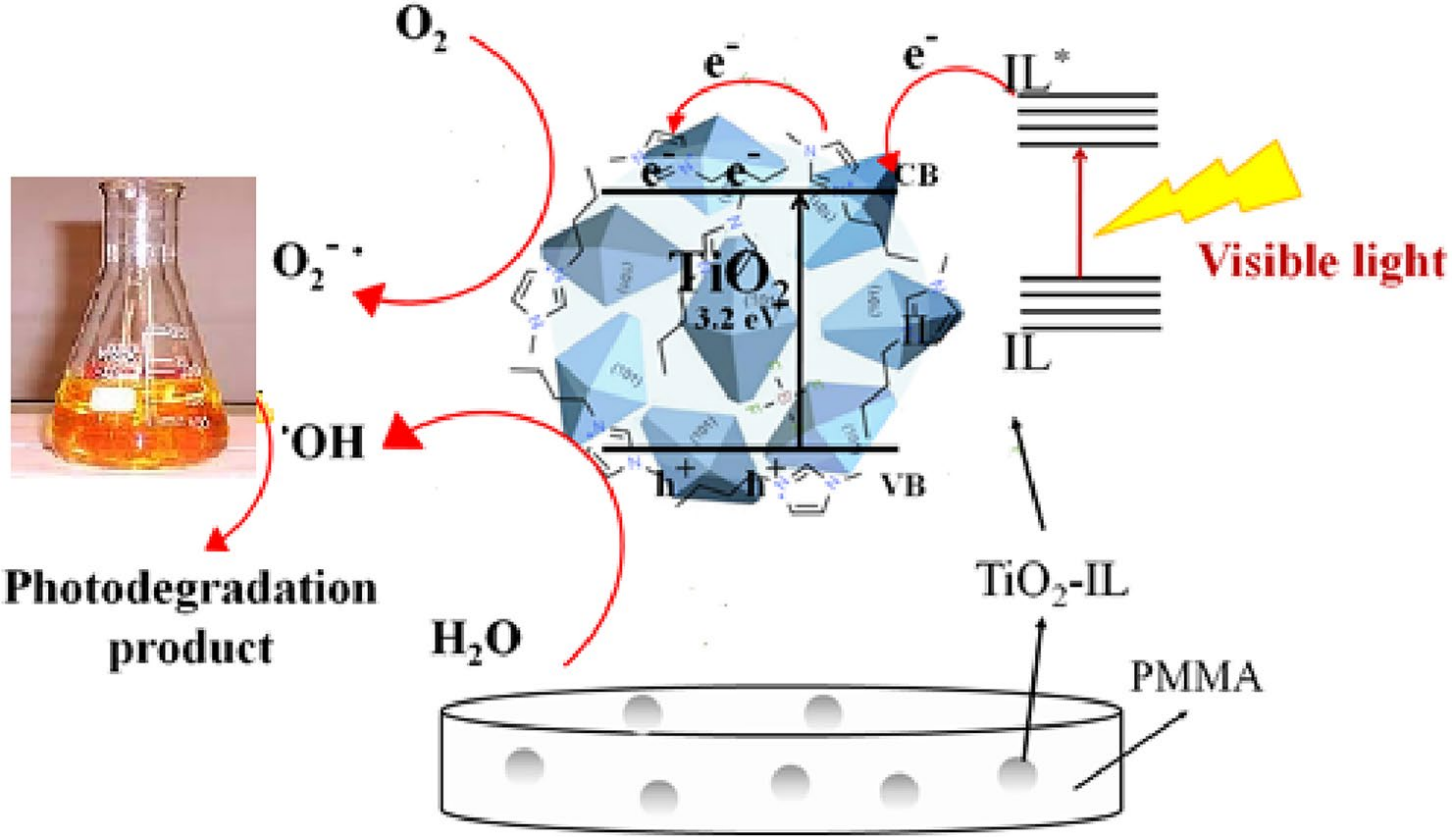
Schematic diagram showing the MO photocatalytic degradation over PMMA/TiO_2_/IL nanocomposite under visible light irradiation.

Figure 9 shows the schematic diagram for the MO photocatalytic degradation over PMMA/TiO_2_/IL nanocomposite which is almost same as the mechanism proposed previously^13^. In this mechanism, the IL molecule excited by absorption of visible light (IL^*^) and then excited IL injects electrons into the conduction band (CB) of the TiO_2_ and is converted into IL^•+^. The photo-generated electrons aid in producing reactive species and would react with adsorbed O_2_ to form ^•^O_2_. However, the leaving holes in the valence band of ionic liquid sensitized TiO_2_ would react with adsorbed OH or water molecule to form ^•^OH. Next, degradation of MO molecules started by the generated of ^•^O_2_, ^•^OH and h^+^.

### Effect of pH on the photodegradation of MO

The effect of pH, as an important variable parameter in aqueous phase mediated photocatalytic reactions, on the MO photodegradation was examined by varying the initial pH of the solution within the range of 5–10 with retaining all other conditions constant. For this purpose, the S2 sample that showed the highest MO degradation rate (at natural pH) in previous section was used and the results collected in Table 2. The best pH value for the MO degradation is obtained around 7. As can be seen from reported data in Table 2, either increases or decreases pH value from 7, the photocatalytic performance will decrease significantly. Actually, change in the pH value plays a critical role in the adsorption of dye molecules on the surface of the photocatalyst and then photodegradation reaction. In other words, change in the pH value might change surface charge of the photocatalyst or charge of the dye molecules and then affecting interaction between dye molecules and nanocomposite surface^28^. Photodegradation of anionic MO dye, that has negatively charged groups due to the presence of the sulfuric group in its structure, better attraction of MO molecules onto the film nanocomposite surface will be occurred at pH = 7.

**Table 2:** The photodegradation percentage and first order rate constants of MO photodegradation reaction over PMMA/TiO_2_/IL at different pH values.

pH	$k\times {10}^{-3}$(min^−1^)	R^2^	Photodegradation of MO after 360 min (%)
5.0	3.4	0.9778	73.3
6.5	4.7	0.9746	83.9
7.0	5.1	0.9552	93.9
8.0	2.4	0.9831	58.7
9.0	1.1	0.9750	31.3
10.0	1.1	0.9397	30.6

### Recycling and photocatalyst stability

**Figure 10:**
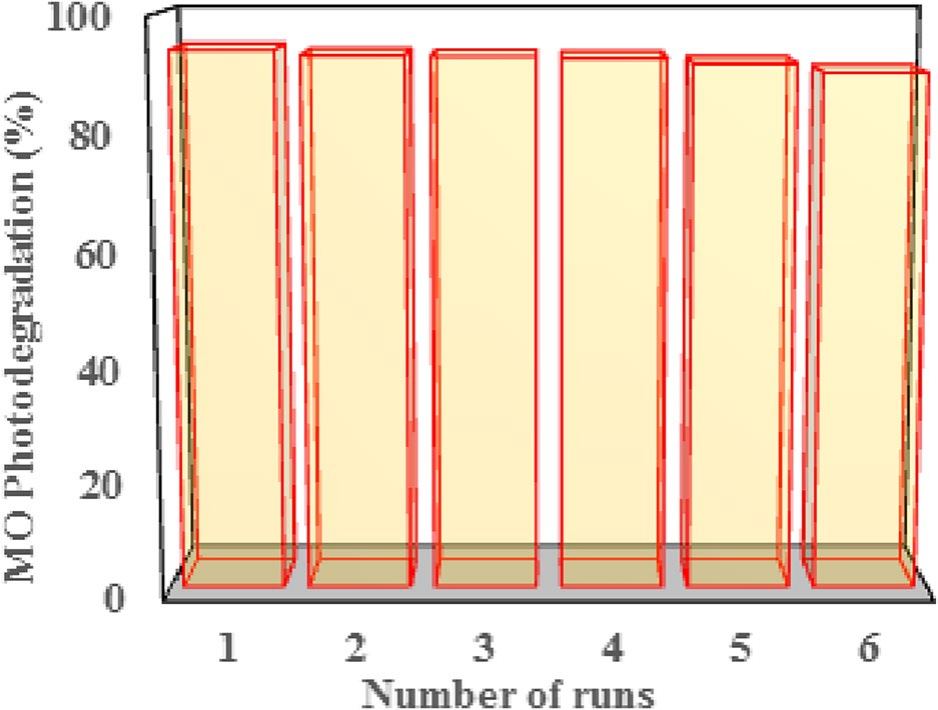
Reusability test of the PMMA/TiO_2_/IL (S2) nanocomposite at pH = 7 after six successive cycles.

Recently, polymer-supported visible light active photocatalyst nanocomposites emerged as a powerful tool used in the wastewater treatment field. The growing interest in this type of nanocomposites is justified by their stability and reusability compared to powder samples that need costly (both time and money consuming) post-treatment separation processes and unfavorable human health risks. Powder sample leads to loss of catalyst mass and as well as photocatalytic activity. In this part, reusability of the prepared nanocomposite for MO decolorization has been investigated. For this purpose, the recycling and stability of the S2 nanocomposite sample was tested under similar circumstances and optimal pH, through successive 6 runs of photocatalyst experiment. After each run nanocomposite was separated and used immediately for further run without any treatment. As can be seen from Fig. 10, the results confirmed that the photocatalyst stability slightly dropped in runs 2 to 6, and producing about 90% degradation. Thus, the prepared nanocomposite is stable when using as visible light photocatalyst and can be employed for degradation purposes more than 6 cycles.

The polymer/TiO_2_ nanocomposites prepared in this work are comparable in efficiency for dye photodegradation to other reported polymer nanocomposites. Sabir et al. recently introduced a polymer nanocomposite containing TiO_2_/Ag nanoparticles^29^. The absorption of TiO_2_ can be shifted towards the visible portion of sunlight by doping with Ag. They reported maximum 81.4% photodegradation efficiency on MO under Xenon light after 180 min. They also examined recyclability with 97% efficiency after 10 cycles. Similar researches have been conducted by Lee et al. on the preparation of a polydimethylsiloxane/Au/TiO_2_ nanocomposite for the photodegradation of Rhodamine B^30^, and by Balarabe et al. on the preparation of a zein polymer/Au/TiO2 nanocomposite for the photodegradation of organic dyes under visible light irradiation^31^. In the present research, for the first time, a facile and effective technique using a surfactant-free microemulsion containing ionic liquid was utilized to immobilize visible-light activated TiO_2_ nanoparticles at low concentrations for MO photodegradation.

## Conclusion

In this research work, it is confirmed that the polymer nanocomposite type of PMMA/TiO_2_/IL, as visible light responsive photocatalyst with high photocatalytic activity, can be prepared using a surfactant-free microemulsion (SFME). This microemulsion system containing methyl methacrylate as oil phase, 1-butanol as amphi-solvent and ionic liquid of [bmim][BF_4_] as dispersed phase. The analysis of the prepared nanocomposite with DRS, (ATR-FTIR) spectroscopy, AFM, and FE-SEM/EDX method confirmed that this preparation method is comparable with surfactant based microemulsion method, and the IL along with amphi-solvent (instead of surfactant) in SFME system creates fine interaction with adsorbed IL molecules on the surface of TiO_2_ nanoparticles to have good compatibility with PMMA. The optimal nanocomposite composition for MO photocatalytic degradation under visible light illumination was determined. In this new preparation method, the suitable bandgap improve the visible-light photocatalytic activity of TiO_2_ nanoparticles in nanocomposite for degradation reaction. The PMMA/TiO_2_/IL nanocomposite sample (S2) demonstrated the highest photocatalytic activity at solution pH of around 7. Moreover, stability and possible degradation mechanism for (S2) nanocomposite were also verified, and the S2 sample exhibited high photocatalytic stability after six cycle's reactions, with no significant decreases in the photocatalytic degradation. This eco-friendly surfactant-free microemulsion preparation is anticipated to provide a new approach and pathway of designing a novel polymer nanocomposite for application in visible light photocatalytic degradation reactions.

## Data Availability

The data are available from the corresponding author upon reasonable request.
